# Herbicidal secondary metabolites from *Bacillus velezensis* JTB8-2 against *Orobanche aegyptiaca*

**DOI:** 10.1186/s13568-022-01395-w

**Published:** 2022-05-07

**Authors:** Wei He, Yan Li, Wenfang Luo, Junhui Zhou, Sifeng Zhao, Jianjun Xu

**Affiliations:** 1 Xinjiang Production and Construction Corps, Key Laboratory of Special Fruits and Vegetables Cultivation Physiology and Germplasm Resources Utilization, Key Laboratory, Universities of Xinjiang Uygur Autonomous Region for Oasis Agricultural Pest Management and Plant Protection Resource Utilization, Agriculture College of Shihezi University, Shihezi, 832003 China; 2 Key Laboratory of Integrated Pest Management on Crops in Northwestern Oasis Ministry of Agriculture, Institute of Plant Protection, Xinjiang Academy of Agricultural Sciences, Xinjiang Urumqi, 830091 China; 3 Institute of Vegetables and Flowers, Chinese Academy of Agricultural Sciences, Beijing, 100081 China

**Keywords:** *Bacillus velezensis*, *Orobanche aegyptiaca*, Secondary metabolites, Seed germination inhibitors, Microbial herbicide

## Abstract

Egyptian broomrape (*Orobanche aegyptiaca*) is a parasitic plants that cause significant losses to important crops. The effective methods for controlling this weed are rare. Biological control could be one of the possible strategies to tackle these weeds efficiently. In this work, a bacteria strain *Bacillus velezensis* JTB8–2 was proven to possesse biological control functions against broomrapes in both pot and field experiments. Four secondary metabolites (**1**–**4**) were isolated from the *B. velezensis* JTB8–2 crude extracts, and all of them could inhibit the germination of *O. aegyptiaca* seeds at concentrations from 0.5 mM to 4 mM. Their structures were further elucidated by Nuclear Magnetic Resonance (NMR) and Mass Spectrometry (MS) analysis. Among the isolated compounds, **1** and **2** exhibited the strongest herbicidal activity with 100% inhibition rate against the germination of *O. aegyptiaca* seeds at 4 mM, and thus had great potential in the development of new herbicidal products to control *O. aegyptiaca* in the future.

**Supplementary Information:**

The online version contains supplementary material available at 10.1186/s13568-022-01395-w.

## Introduction

The broomrape (*Orobanche* spp.) is an obligate holoparasitic weed without functional photosynthetic system that causes severe damage to vegetables and field crops worldwide. It relies on a unique organ, the “haustorium”, to intercept water and nutrients from the host to maintain its growth, which affects the growth of the host and causes serious losses to the yield and quality of crops. Egyptian broomrape (*Orobanche aegyptiaca*) is a widespread parasitic weed of many *Solanaceae*, *Brassicaceae*, *Cannabaceae*, *Fabaceae*, *Apiaceae*, *Asteraceae*, and *Curcurbitaceae* species (Eizenberg and Goldwasser 2018). It is distributed mainly in the Mediterranean area, North Africa, and Asia (Boari and Vurro et al. 2004; Qasem 1998; Zehhar et al. 2002). Xinjiang is the region with the most extensive distribution and serious damage of *O. aegyptiaca* in China, and it had extensive infestations of muskmelon, watermelon and processing tomato, leading to 20–70% yield losses in Xinjiang Province, China (Parker 2009). The underground seed germination and parasitism activities of this parasitic weed are not easily detectable, consequently making it difficult to control. When the parasite finally emerges above ground, the majority of the damage to the host has already been done, and control would be ineffective.

The currently available methods to control broomrape include trap crops (Qasem 2019), hand weeding, soil solarization (Habimana et al. 2014; Mauromicale et al. 2005); soil fumigation and use of chemical herbicides (Eizenberg and Goldwasser 2018). In most cases, these approaches are time-consuming, hazardous to the environment, and mostly inefficient. Microorganisms are an environmentally friendly and effective tool to reduce broomrape infestation in cropping systems, such as *Pseudomonas* spp. and *Bacillus* spp. (Zermane et al. 2007; Barghouthi et al. 2010). Microorganisms include symbionts (e.g., *Rhizobium* spp.) which colonize root of host plants and non-symbionts (e.g., *Alternaria* spp. and *Fusarium* spp.) El-Halmouch et al. 2013; de Zélicourt et al. 2009) which directly attack the parasite. In addition, microorganism metabolites can also inhibit seeds germination and thus reduce broomrape seed banks, such as *Myrothecium verrucaria*, *Fusarium compactum* and *Pleurotus ostreatus*. (EI-Kassas et al. 2005; Andolfi et al. 2005; Elsakhawy et al. 2020). This is a biological method which can be included in an integrated broomrape management program. Strategies for controlling parasitic weeds by using natural products include inducing “suicide germination”. The ability of both artificial and natural compounds to induce germination of parasitic seeds was investigated. Analogues of strigolactone have been synthesized, such as GR24 and Nijmegen 1, which can effectively induce the germination of broomrape seeds (Wigchert et al. 1999). However, their poor stability in soil and the high cost of producing these compounds limit their use in agricultural production (Babiker et al. 1987; Dvorakova et al. 2019) synthesized simple and stable strigolactone mimics with selective activity against *Phelipanche ramosa*. Kuruma et al. (2021) discovered a structurally hybrid compound that can induce spontaneous germination and inhibit subsequent radical growth. However, their stability in soil and toxicology remains unclear. Other natural compounds, including mycotoxins (Evidente et al. 2006), coumarins (Serghini et al. 2001) and methyl jasmonate (Yoneyama et al. 1998), have been shown to induce the germination of witchweed and broomrape seeds, but their potential applications in agriculture need further investigation.

The use of microbial secondary metabolites to research and develop natural herbicides with high activity, high selectivity, and high safety will be an important direction for the development of new herbicides. *B. velezensis*, as a novel species, is harmless to human and animal, and its metabolites include antibiotic lipopeptide, polyketides and peptide (Ye et al. 2018). At present, the biocontrol effect of *B. velezensis* is mainly focused on plant diseases Nifakos et al. 2021; Cheffi et al. 2019; Zhang et al. 2021). In this paper, a bacteria strain *Bacillus velezensis* JTB8–2 is proven to possess biological control functions of Egyptian broomrape in tomato pot and in the processing tomato field. Four pure herbicidal secondary metabolites are isolated from this strain, and detailed of their chemical purification, structure identification, and the inhibitory effects on the germination of *O. aegyptiaca* seeds are described.

## Materials and methods

### Origin of strains

Strain JTB8–2 was isolated from the rhizosphere soil of Egyptian broomrape in Jimusar County, Xinjiang Province, China, and was identified as *B. velezensis*. The strain was also deposited in the Guangdong Microbial Culture Collection Center (No. GDMCC 60,755). This strain was grown on Nutrient Agar (NA) medium (3 g beef paste, 7 g peptone, 5 g NaCl, and 17 g agar in 1 L distilled water, pH 7.0) for routine culturing.

### Fermentation

The purified single colony was inoculated into NB medium (3 g beef paste, 7 g peptone, 5 g NaCl in 1 L distilled water, pH 7.0), and the bacterial culture was obtained through incubation at 130 revolutions per minute (rpm) at 25 ℃ for 24 h. The bacterial culture was transferred into two 500 mL-Erlenmeyer flasks containing 200 mL NB medium, and fermentation broth was obtained by oscillating culture at 28 ºC and 180 rpm for 24 h.

### Pot experiment

The experimental treatments included strain JTB8-2 fermentation broth, culture medium NB and blank control, with 3 replicates per treatment and 3 pots per replicate. Plastic basin specifications were 25 cm in diameter and 18 cm in height, with holes at the bottom. Tomato cultivar HS1015 was planted in hole trays and transplanted when tomato seedlings had 4 ~ 5 leaves. Potting soil was medium loam soil (Alkaline hydrolyzed nitrogen: 82.682 mg/kg; Available phosphorus: 42.824 mg/kg; Available potassium: 289.522 mg/kg; pH: 7.86) which taken from the field of the Anning Ditch test site in Urumqi, Xinjiang. About 200 g of fine soil was put into a 600 mL plastic bottle and then added 50 mg (approximately 10,000 seeds) *O. aegyptiaca* seeds, the mixture was shaken well and sprinkled evenly on the tomato roots. Covered with soil and then poured water. *B. velezensis* JTB8-2 fermentation broth at a concentration of 5 × 10^8^ CFU/mL and culture medium NB were diluted 25-fold, 50-fold, 100-fold respectively, and then irrigated with 1 L/pot the next day, once every 15 d, 3 consecutive times, and water as blank control. Tomato root soil was removed 60 d after transplantation, and the parasitism number in each pot was investigated. The fresh weight was taken, and then the collected broomrape was put into the oven to dry at 60 °C.

### The field test

Field experiments were carried out on processing tomato in Shuanghe Village, Qingyang Lake Township, Jimusar County, Changji Prefecture, Xinjiang (E: 89°1’ 35”; N: 44°2’ 3”). The soil was medium loam soil (Alkaline hydrolyzed nitrogen: 80.627 mg/kg; Available phosphorus: 41.033 mg/kg; Available potassium: 237.655 mg/kg; pH: 7.88). The experimental treatments included JTB8–2 agent 0.8 L/105m^2^, 1.6 L/105m^2^, 3.2 L/105m^2^ and water, with three replicates per treatment. Each replicate was randomly arranged in the field. The fermentation broth concentration of the strain was 5 × 10^8^ CFU/mL by turbidimetric method. The first drip irrigation, 15 d after tomato seedlings were transplanted, came from an electric sprayer, and 40 L of bacterial liquid was applied every time, applied once every 20 d on 3 consecutive times. The first field investigation was carried out 25 d after the third application. 30 m^2^ area was select randomly from each 105 m^2^ area respectively, andthe number of *O. aegyptiaca*, tomato plants, and broomrape parasites were investigated and fresh weight was taken. The collected broomrape was placed into an oven to dry at 60 °C, and the biomass was collected.

$$\begin{gathered} {\text{Parasitism rate }}\left( \% \right)\, = \,{\text{number of parasitized tomato plants}}/{\text{number of investigated }} \hfill \\ {\text{tomato plants}}\, \times \,{1}00. \hfill \\ \end{gathered}$$

The measured yield area of each plot was 4.5 m^2^. Yield increasing effects of bacterial treatments were evaluated by determining fruit weight of plant (kg/plant), weight of 100 fruits (kg/100) and fruit weight of plot (kg).

### Chemical extraction, isolation and purification

The scaled-up fermentation was carried out in ten 1000 mL-Erlenmeyer flasks. For each flask, 10 mL secondary seed fermentation liquid was inoculated into 200 mL NBmedium, and 9 g macroporous resin (XAD-16) was added to absorb the secreted metabolites. After incubation at 28 °C for 5 days, the fermentation broth was discarded, and the macroporous resin was repeatedly washed with distilled water and dried in oven at 28 ºC. Next, the resin was extracted with 300 mL methanol for 3 times, and the resulting methanol solution was combined and concentrated under vacuum. The concentrate was redissolved in 150 mL 50% methanol solution, and then extracted with equal-volume dichloromethane for 4 times. The organic layer was evaporated to dryness under vacuum to get a 6.5 g crude extract, which was further fractionated by silica gel Vacuum Liquid Chromatography (VLC) eluting with gradient PE (petroleum ether)–EtOAc (Ethyl acetate) solution. All fractions were evaluated for their inhibitory effects on the germination of Egyptian broomrape seeds, and inhibition rate of fractions eluted with 25% and 40% EtOAc were 100%. Thus, the 25% EtOAc fraction (87.68 mg) was purified by reverse phase high performance liquid chromatography (RP HPLC) on a C18 column (Kromasil 100-5-C18; 5 μm; 10 × 250 mm; 40% MeOH in H_2_O over 28 min; 2 mL/min) to get compound **1** (6.1 mg, *t*_R_ 9.10 min) and **2** (20 mg, *t*_R_ 11.99 min). The 40% EtOAc fraction (153.1 mg) was also purified by semipreparative RP HPLC (40% MeOH in H_2_O over 30.0 min; 2 mL/min) to get compounds **3** (4.5 mg, *t*_R_ 13.4 min) and **4** (7.0 mg, *t*_R_ 24.5 min).

### NMR analysis

The ^1^ H and ^13^ C NMR data were collected on a Bruker Avance 500 MHz NMR spectrometer equipped with a 5-mm triple resonance cryoprobe at 298 K. Chemical shift values (*δ*) are given in parts per million (ppm) and the coupling constants (*J* values) are in Hz. Chemical shifts were referenced to the residual solvent peaks.

### Bioactivity assay

Seed sterilization: The *O. aegyptiaca* seeds were disinfected in 75% ethanol for 30 s and then transferred to 3% sodium hypochlorite (effective chlorine) solution for 10 min. After rinsing in sterilized water 3 times, the seeds were dried on sterile filter paper for later use.

Activity determination of crude extract: 100 mg crude extract was dissolved in 100 µL methanol and then mother liquor was prepared with add 100 µL distilled water for activity test before column chromatography separation. Whatman filter paper (GF/A) was cut into round paper with a diameter of 14 mm and placed in a 24-well cell culture plate bottom, with 2 pieces for each well. Approximately 50 sterilized and dried seeds were added to each well. Dissolved 1 mg GR24 in 200 mL distilled water. Added 200 µL GR24 (5 µg/mL) solution to each well and then added the crude extract mother liquor, respectively. Crude extracts were assayed at a final concentration from 5 µg/mL to 0.6 µg/mL. Water was set as blank control. After 5 days of shading culture at 25 °C, the number of germinated seeds was observed under a microscope. The germination rate and inhibition rate were calculated according to the following formula for evaluate the effect of crude extract. The assay was repeated 4 times for each concentration.

$${\text{Germination rate }}\left( \% \right) \, = {\text{ Germination seed number}}/{\text{The total number of seeds}} \times {1}00$$

$$\begin{gathered} {\text{Inhibition ratio }}\left( \% \right)\; = \;({\text{Seed germination rate of blank control}}{-}{\text{Germination rate of treated seeds}}) \hfill \\ /{\text{Seed germination rate of blank control}} \times {1}00 \hfill \\ \end{gathered}$$

Activity determination of pure compounds: Nitrogen blowing was carried out on the nuclear magnetic tube solution, and the quality of each compound was detected after drying. According to the molecular weight of different compounds, 8 mM mother liquor was prepared by adding sterile water and then diluted to 4 mM, 2 mM and 1 mM. Then, 0.25 mL of the assay solution was mixed with 0.25 mL GR24 solution at a concentration of 5 µg/mL. The pure metabolites were assayed at concentrations between 4 mM and 0.5 mM. The assay was repeated 4 times for each pure compound.

### Statistical analysis

Data were presented as mean ± SE and analyzed using analysis of variance. For *in vitro* and *in vivo* results, completely randomized design (CRD) was used while field data were analyzed using randomized complete block design (RCBD). Statistical software SPSS 16.0 was used. Significant difference in the treatment was measured with LSD (Least Significant Difference) test and separated by using lettering.

## Results

### Control effect of *B. velezensis* JTB8–2 on* O. aegyptiaca* in pot experiment

The results of pot experiment showed that treatments of neither *B. velezensis* JTB8–2 fermentation broth nor NB medium in three different dilutions had substantial impact on the parasitic rates of *O. aegyptiaca* as compared to the control (Table 1). However, treatments of *B. velezensis* JTB8–2 fermentation broth at 25-fold, 50-fold and 100-fold dilutions largely reduced the fresh weight and biomass of *O. aegyptiaca*, while no significant differences in *O. aegyptiaca* fresh weight and biomass were observed between the NB medium treatment and the control (Table 1). Moreover, the biomass of *O. aegyptiaca* after the treatment of *B. velezensis* JTB8–2 fermentation broth at 25-fold dilution (1.82 g/pot) was significantly lower than that of the same treatment at 100-fold dilution (3.88 g/pot) (Table 1). These results indicated that *B. velezensis* JTB8–2 could control the growth of *O. aegyptiaca* under potted condition.

**Table 1:** Parasitic rate, *O. aegyptiaca* number, fresh weight and biomass of different treatment in pot experiment

Treatment	Diluted multiples	Parasitic rate (%)	Number (Shoots/pot)	Fresh weight (g/pot)	Biomass (g/pot)
JTB8–2	25-fold	100	4.33 ± 0.62^b^	14.42 ± 1.41^c^	1.82 ± 0.17^c^
	50-fold	100	6.33 ± 0.99^b^	21.97 ± 2.73^b^	2.78 ± 0.34^b^
	100-fold	100	11.11 ± 1.18^a^	29.78 ± 3.31^b^	3.88 ± 0.47^b^
NB	25-fold	100	15.78 ± 1.51^a^	44.72 ± 2.63^a^	5.68 ± 0.33^a^
	50-fold	100	15.33 ± 1.69^a^	45.07 ± 3.10^a^	5.67 ± 0.38^a^
	100-fold	100	15.44 ± 1.73^a^	45.59 ± 3.37^a^	5.87 ± 0.42^a^
CK	Water	100	15.22 ± 1.81^a^	45.40 ± 5.69^a^	5.85 ± 0.75^a^

The results of variance analysis showed significant differences between different treatments, the different small letters represent significance at 5% level

### Control of effect of B. velezensis JTB8–2 on O. aegyptiaca in the field

**Fig. 1:**
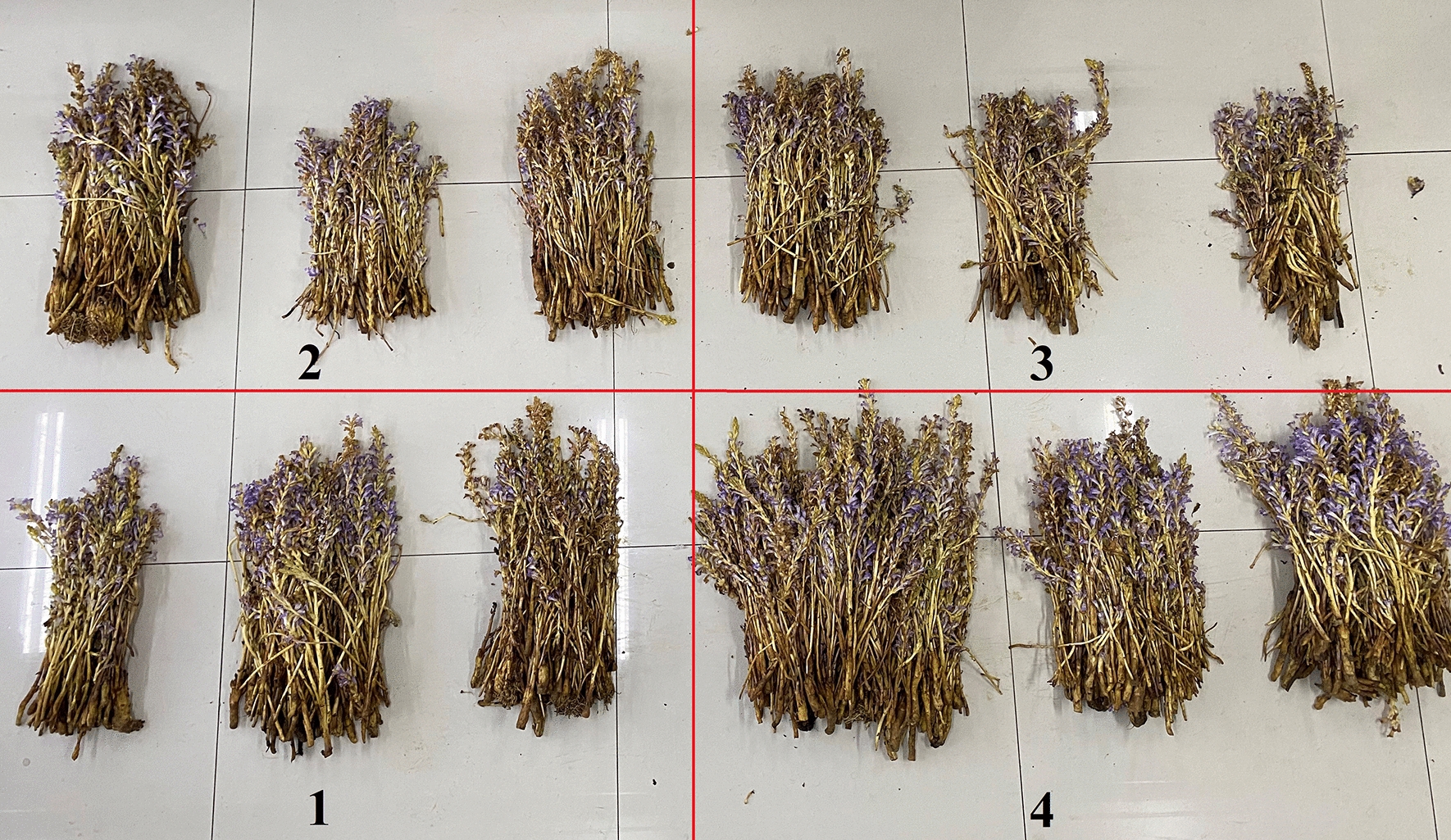
The Egyptian broomrape number of different treatments in the field. **1** was JTB8–2 agent 0.8 L/105m^2^; **2** was JTB8–2 agent 1.6 L/105m^2^; **3** was JTB8–2 agent 3.2 L/105m^2^; **4** was blank control

Next, we evaluated the control effect of *B. velezensis* JTB8–2 on *O. aegyptiaca* in the field by applying the fermentation broth of *B. velezensis* JTB8–2 at three different dosages (0.8, 1.6 and 3.2 L/105m^2^ in treatments 1, 2 and 3, respectively). All three treatments were able to reduce the parasitic rate and the number of shoots of *O. aegyptiaca* (Table 2; Fig. 1), and treatment 3 displayed the strongest effect (6.76% parasitic rate and 41.67 shoots/30m^2^). Additionally, treatment 3 also reduced the fresh weight and biomass of *O. aegyptiaca* by 65% as compared to the control, while the other two treatments were less effective (Table 2). This suggested that the *B. velezensis* JTB8–2 could control the growth of Egyptian broomrapes in the field.

**Table 2:** Parasitic rate, *O. aegyptiaca* number, fresh weight and biomass of different treatment in the field

Treatment	Parasitic rate (%)	*O. aegyptiaca* number (Shoots/30m^2^)	Fresh weight (g/30m^2^)	Biomass (g/30m^2^)
1	16.58 ± 2.5^b^	102.33 ± 8.09^a^	2000 ± 357.26^b^	267.64 ± 58.55^b^
2	10.53 ± 2.95^c^	64.33 ± 5.36^b^	1936.67 ± 417.03^b^	256.05 ± 50.93^b^
3	6.76 ± 0.83^d^	41.67 ± 4.91^c^	1476.67 ± 322.71^c^	190.13 ± 38.44^c^
CK	21.76 ± 4.30^a^	114.67 ± 8.11^a^	4236.67 ± 834.11^a^	540.66 ± 85.51^a^

**1** was JTB8-2 agent 0.8 L/105m^2^; **2** was JTB8-2 agent 1.6 L/105m^2^; **3** was JTB8-2 agent 3.2 L/105m^2^; CK was water. The different small letters represent significance at 5% level

### Effects of B. velezensis JTB8–2 on the growth and fruiting of tomato

**Fig. 2:**
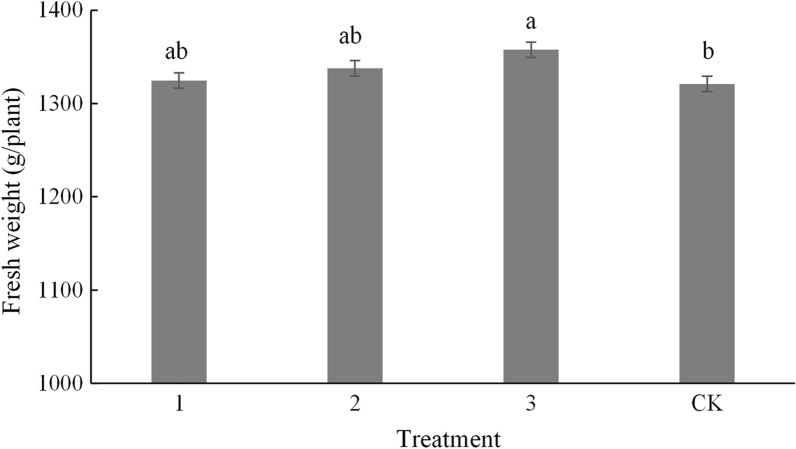
The tomato plant fresh weight of different treatments in the field. **1** was JTB8–2 agent 0.8 L/105m^2^; **2** was JTB8–2 agent 1.6 L/105m^2^; **3** was JTB8–2 agent 3.2 L/105m^2^; CK was blank control

To investigate the impact of *B. velezensis* JTB8–2 on the growth and fruiting of crops, tomato plants were treated with the fermentation broth of *B. velezensis* JTB8–2 in the same way as Egyptian broomrapes. The results showed that all three treatments increased the fresh weight of tomato plant with treatment 3 being most effective (Fig. 2). Moreover, applying the fermentation broth of *B. velezensis* JTB8–2 significantly increased the fruit weight of plant and the fruit weight of plot by more than 0.8 kg/plant and 7.2 kg/plant, respectively (Table 3). However, no significant differences were found among all three treatments and the control concerning the weight of 100 fruits (Table 3). Taken together, these results demonstrated that *B. velezensis* JTB8–2 could promote the growth of tomato plant and increase the yield of fruits.

**Table 3:** Fruit weight of plant, weight of 100 fruits and fruit weight of plot in the field

Treatment	Fruit weight o plant (kg/plant)	Weight of 100 fruits (kg/100)	Fruit weight of plot (kg)
1	4.03 ± 0.11^a^	5.38 ± 0.13^a^	65.75 ± 1.72b^c^
2	4.03 ± 0.18^a^	5.65 ± 0.22^a^	68.19 ± 1.52^ab^
3	4.02 ± 0.19^a^	5.58 ± 0.12^a^	72.15 ± 1.02^a^
CK	3.19 ± 0.19^b^	5.18 ± 0.12^a^	58.49 ± 2.23^c^

**1** was JTB8-2 agent 0.8 L/105m^2^; **2** was JTB8-2 agent 1.6 L/105m^2^; **3** was JTB8-2 agent 3.2 L/105m^2^; CK was water. The different small letters represent significance at 5% level

### Bioactivities of extracts from B. velezensis JTB8–2 fermentation broth

**Fig. 3:**
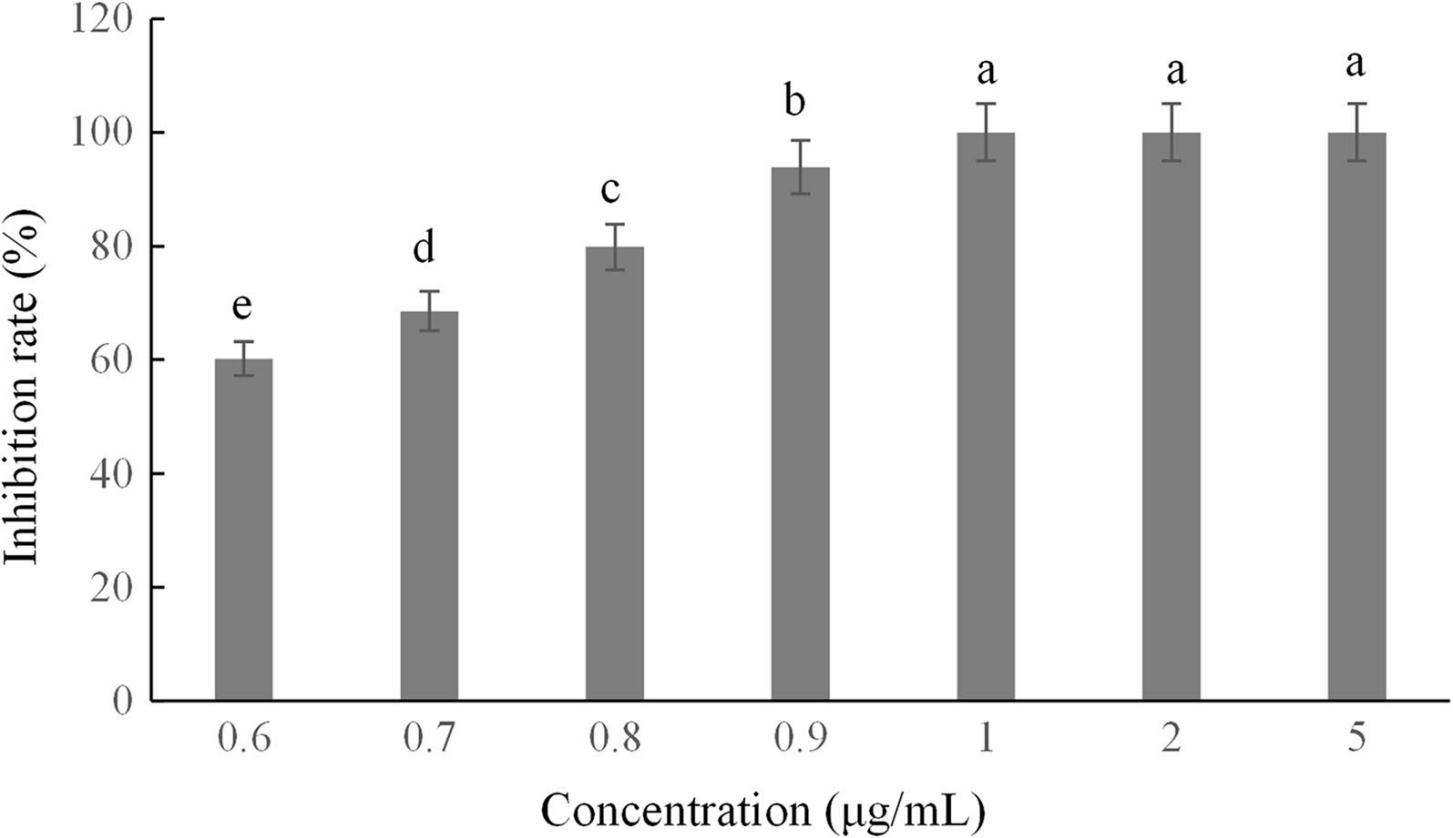
The germination rate and inhibition rate of *O. aegyptiaca* seeds treated by different concentration of crude extract from *B. velezensis.* The different small letters represent significance at 5% level

The crude extracts from *B. velezensis* JTB8–2 fermentation broth significantly inhibited the germination of Egyptian broomrape seeds in a dose-dependent manner. The treatments of 1, 2 and 5 µg/mL crude extracts were most effective which showed 100% inhibition rate (Fig. 3). This result strongly indicated that the crude extracts contain substances with potential herbicidal activity against Egyptian broomrape. Subsequently, bioactivity-guided separation of the crude extracts using silica gel VLC resulted in two active fractions (eluted with 25% and 40% EtOAc, respectively), from which compounds **1**–**4** were isolated.

### Structure elucidation

**Fig. 4:**
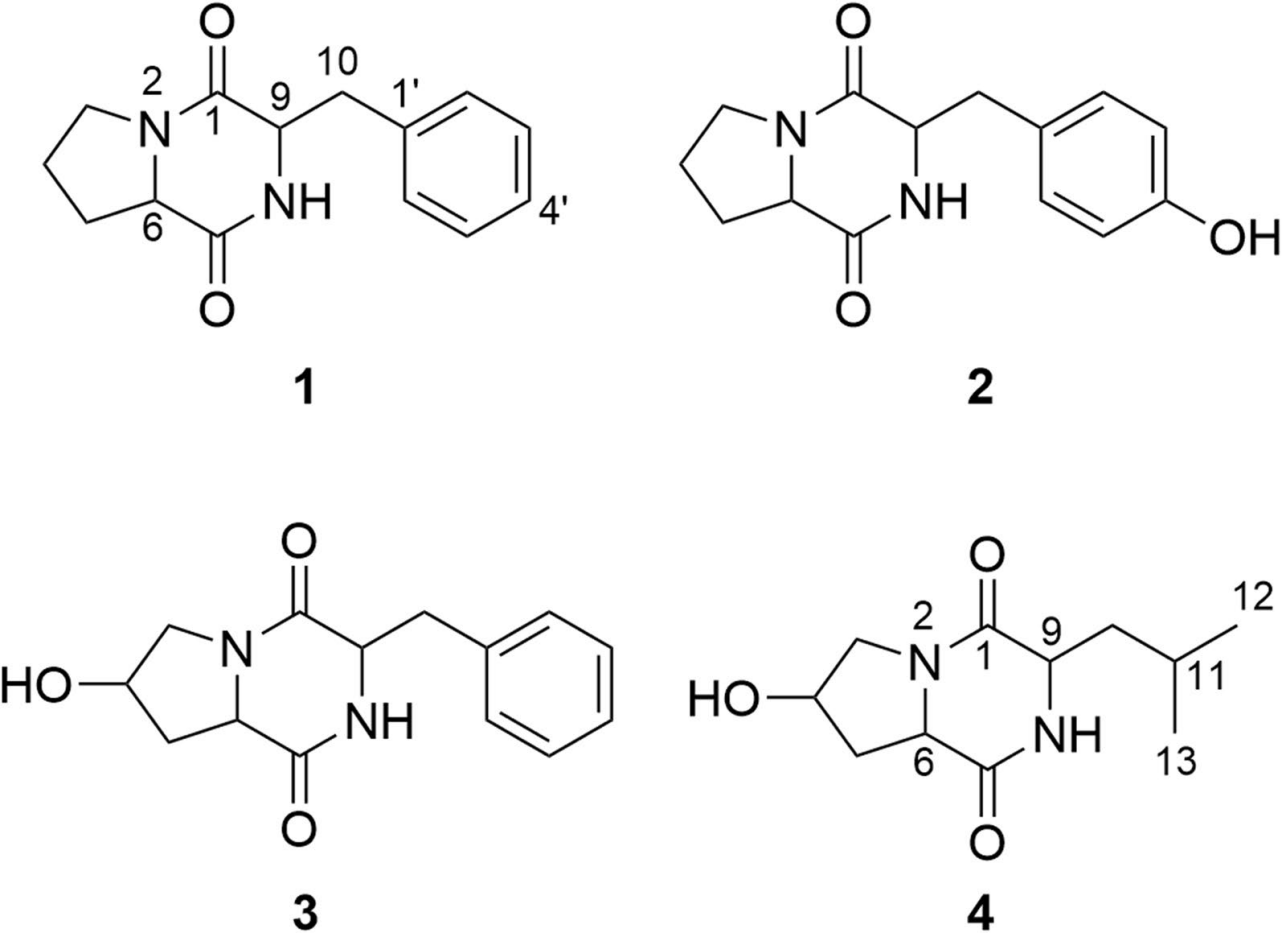
Chemical structures of compounds **1**–**4**

The positive mode ESI-MS spectrum of **1** exhibited an [M + H]^+^ ion at *m*/*z* 245.0 (Additional file 1: Fig. S1). The presence of characteristic ^13^ C NMR signals for the amide carbonyl groups (*δ*_C_ 169.9 and 165.9), as well as the ^1^ H and ^13^ C NMR chemical shifts for heteroatom-bounding methine functionalities (*δ*_H_ 4.38 and 4.12; *δ*_C_ 59.6 and 57.1) suggested that compound **1** has a diketopiperazine scaffold which is consisted of two amino acid moieties (Tables 4 and 5). One of the amino acid unit was deduced as phenylalanine (Phe) based on the presence of resonance signals corresponding to a monosubstituted benzene ring (*δ*_H_ 7.23–7.35; *δ*_C_ 127.5–138.2) and a slightly de-shielded methylene functionality (*δ*_H_ 3.02 and 3.33; *δ*_C_ 37.0). The remaining ^1^ H and ^13^ C NMR signals for another three methylene groups (Tables 4 and 5) indicated that the other amino acid is proline (Pro). Thus, the planar structure of **1** was determined as cyclo-(Pro-Phe) (**1**; Fig. 4) and its ^1^ H and ^13^ C NMR data were in agreement with those found in the literature (Jayatilake et al. 1996). The positive mode ESI-MS spectra of **2** and **3** both showed an [M + H]^+^ ions at *m*/*z* 261.0 (Additional file 1: Figs. S2–3), indicating they share the same molecular weight. Moreover, the ^1^ H and ^13^ C NMR data of **2** and **3** were very similar to that of **1** except for considerable variations on the proline or benzene ring (Tables 4 and 5). Comparison of these data with those reported in literature (Jayatilake et al. 1996; Fdhila et al. 2003) confirmed the structures of **2** and **3** as cyclo-(Pro-Tyr) and cyclo-(4-OH-Pro-Phe), respectively (**2** and **3**; Fig. 4). An [M + Na]^+^ ion was found at *m*/*z* 249.0 in the positive mode ESI-MS spectrum of **4** (Additional file 1: Fig. S4). Analysis of the ^1^ H and ^13^ C NMR data (Tables 4 and 5) revealed that **4** adopts a leucine (Leu) unit in place of the Phe moiety found in the structures of **3**. Comparison of these data with those reported by Shigemori et al. (1998) established the structure of **4** as cyclo-(4-OH-Pro-Leu) (**4**; Fig. 4).

**Table 4:** ^1^H NMR data (500 MHz) of compounds **1**–**4**

Proton	**1**^***a***^	**2**^***b***^	**3**^***a***^	**4**^***a***^
	*δ*_H_, mult. (*J* in Hz)	*δ*_H_, mult. (*J* in Hz)	*δ*_H_, mult. (*J* in Hz)	*δ*_H_, mult. (*J* in Hz)
3	3.53, m	3.55, m	3.60, dd (3.8, 12.1)	3.61, dd (4.5, 12.5)
	3.38, m	3.34, m	3.27, dd (5.9, 12.1)	3.38, dd (2.5, 12.5)
4	1.84, m	1.80, m	3.10, m	4.13, m
5	2.13, m	2.09, m	2.23, m	2.19, m
	1.67, m	1.22, m	1.99, m	2.07, m
6	4.38, t (5.7)	4.36, t (5.5)	4.30, m	4.48, t (4.4)
9	4.12, m	4.05, ddd (2.0, 6.3, 10.9)	4.15, m	4.44, dd (6.7, 10.8)
10	3.33, dd (4.3, 14.1)	3.08, dd (5.2, 14.1)	3.13, dd (5.9, 1.2)	1.97, m
	3.02, dd (6.9, 14.1)	3.02, dd (4.5, 14.1)	3.02, dd (4.7, 13.2)	
11				1.49, m
12				0.95, t (4.2)
13				0.95, t (4.2)
2'	7.35, d (8.0)	7.04, d (8.5)	7.28, m	
3'	7.30, dd (7.5, 8.0)	6.70, d (8.5)	7.21, m	
4'	7.23, d (7.5)		7.28, m	
5'	7.30, dd (7.5, 8.0)	6.70, d (8.5)	7.21, m	
6'	7.35, d (8.0)	7.04, d (8.5)	7.28, m	

^*a*^Acetone-*d*_6_ as solvent

^*b*^Methanol-*d*_4_ as solvent

**Table 5:** ^13^C NMR data (acetone-*d*_6_, 125 MHz) of compounds **1**, **3** and **4**

Carbon	**1**	**3**	**4**
	*δ*_C_, type	*δ*_C_, type	*δ*_C_, type
1	165.9, C	165.9, C	171.3, C
3	45.6, CH_2_	53.8, CH_2_	54.0, CH_2_
4	23.1, CH_2_	68.3, CH	68.8, CH
5	28.9, CH_2_	40.7, CH_2_	38.1, CH_2_
6	59.6, CH	59.4, CH	54.8, CH
7	169.9, C	168.9, C	167.3, C
9	57.1, CH	56.7, CH	58.2, CH
10	37.0, CH_2_	38.1, CH_2_	39.3, CH_2_
11			25.4, CH
12			23.3, CH_3_
13			22.1, CH_3_
1'	138.2, C	137.3, C	
2'	129.2, CH	129.2, CH	
3'	130.6, CH	130.9, CH	
4'	127.5, CH	127.8, CH	
5'	130.6, CH	130.9, CH	
6'	129.2, CH	129.2, CH	

### Inhibition effects on O. aegyptiaca seeds germination of the four pure compounds

**Fig. 5:**
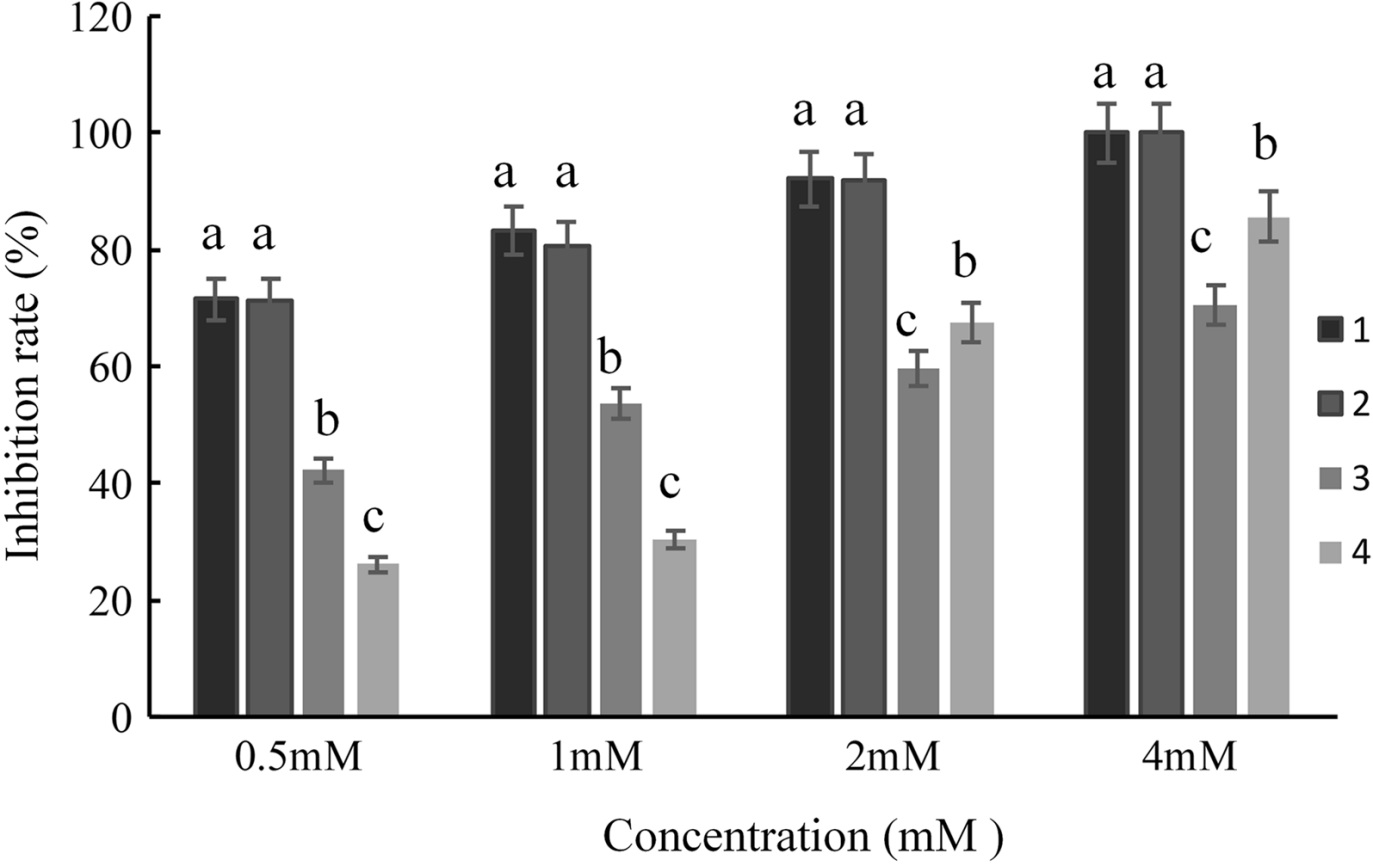
Effect of the compounds **1**–**4** on the percent inhibition of *O. aegyptiaca* seeds germination. The different lowercase letters represented significant difference at 0.05 level

All four pure compounds inhibited *O. aegyptiaca* seed germination, and the inhibitory effects for Compounds **1** and **2** were the best, as they both had a 100% inhibitory effect on *O. aegyptiaca* seeds germination at a concentration of 4 mM (Fig. 5). Meanwhile, Compound **4** also showed over 80% inhibitory effects on *O. aegyptiaca* seeds germination at a concentration of 4 mM, and the inhibitory effects for Compound **3** was the worst, showing a less than 65% at a concentration of 4 mM (Fig. 5).

## Discussion

In recent years, soil microorganisms have been paid more and more attention as an effective biocontrol tool for broomrape (Boari and Vurro 2004; Sauerborn et al. 2007; Zermane et al. 2007; Iasur Kruh et al. 2017). Soil microbiomes can affect broomrape emergence and growth via form biofilms, produce toxins or chang the rhizosphere condition caused by them. For example, Iasur Kruh et al. (2017) suggested that the biocontrol effect of *Pseudomonas* on *Phelipanche aegyptiaca* was attributed to the diverse compounds released by the bacteria which inhibit broomrape and improve the immune system of the host plant. In addition, Compared with other non-soil microorganisms, soil microorganisms can play a role in the early growth stage of broomrape, and will not pollute soil and environment, and easily colonize and reproduce in soil and continue to play a control role. In this study, *B. velezensis* JTB8–2 strain was isolated from a local field soil, and its fermentation broth significantly reduced the fresh weight and dry weight of *O. aegyptiaca* in both pot and field experiments, indicated that the strain has the potential to control *O. aegyptiaca*. however, its colonization and reproductive ability in field soil and biocontrol mechanism remains unclear.

Bacteria have the advantages of fast growth, high antibacterial activity, and environmental friendliness compared with other microorganisms, which makes them a microorganism with great potential to develop microbial pesticides. *B. velezensis* is a new species isolated from marine environments, soil, and plants in recent years, and its biological control effect on a variety of plant diseases have been reported, such as lotus root rot disease (Wang et al. 2019), soybean root rot disease (Huang et al. 2017), and anthracnose (Huang et al. 2017). However, the biological control effect of its metabolites on the inhibition of *O. aegyptiaca* seeds germination is still reported for the first time. In addition, the growth-promoting ability of *B. velezensis* on different crops also has been reported (Chen et al. 2019; Azabou et al. 2020; Torres et al. 2020). Our study also confirmed that the fermentation broth of *B. velezensis* JTB8-2 had the potential to promote tomato plant growth and yield increase. This may be due to *O. aegyptiaca* decrease and its own growth-promoting effect.

It will be one of the important directions to use microbial metabolites and artificial compounds of these metabolites to develop natural herbicides with high activity, high selectivity and high safety. Many reports have shown that the metabolites of microorganisms contain natural substances that inhibit the germination of seeds. Regarding infesting broomrapes, Zonno and Vurro (2002) reported that some toxins produced by fungi of the *Fusarium* genus were able to inhibit the germination of *O. ramosa* seeds and proposed their practical use for parasitic plant management. Louarn et al. (2012) reported that the extract of arbuscular mycorrhizal fungi has a significant inhibitory effect on the germination of *O. cumana* seeds. De Zélicourt et al. (2009) reported that sphinganine-analogue mycotoxins extracted from *Alternaria alternata* have a preventive effect on *Orobanche* spp. Andolfi et al. (2005) reported that 7 toxins isolated from *Fusarium* could inhibit the germination of 100% of *Orobanche* seeds at a concentration of 10 mM. Among them, neosolaniol, diacetoxyscirpenol, T-2, and HT-2 toxins can completely inhibit the germination of *Orobanche* seeds, even at a concentration of 1 mM. It takes a few days to establish parasitism between broomrape seed with the host plant, and the germination time of broomrape seed is not consistent in the soil (Joel 2000). Therefore, metabolites need to be stable in the soil for their continued control function. However, there are no many reports on stability of microorganism metabolites in soil.

The production of compounds **1**–**4** and other diketopiperazine-type metabolites has been investigated in many microorganisms, such as *Pseudomonas aeruginosa* (Jayatilake et al. 1996), *Xenorhabdus nematophila* and *Photorhabdus temperate* subsp. *temperate* (Seo et al. 2012), and *Aureobasidium pullulans* (Shigemori et al. 1998). However, the four metabolites were the first reported inhibitors for the germination of *O. aegyptiaca* seeds, which were isolated from the organic crude extract of *B. velezensis*. The current study demonstrated the potential application of these diketopiperazines as natural herbicides for the management of *O. aegyptiaca* seeds germination. However, their stability in soil and toxicology remains unclear.

## Supplementary Information

**Additional file 1: Figure S1.** (+)-ESI-MS spectrum of compound 1,** Figure S2.** (+)-ESI-MS spectrum of compound 2,** Figure S3.** (+)-ESI-MS spectrum of compound 3,** Figure S4.** (+)-ESI-MS spectrum of compound 4

## Data Availability

The datasets on which the conclusions of the manuscript rely to were presented in the main paper.
