# Supplementary material for: Herbicidal secondary metabolites from Bacillus velezensis JTB8-2 against Orobanche aegyptiaca
Source: AMB Express. 2022 May 7;12:52. doi: 10.1186/s13568-022-01395-w (PMC9079202; doi:10.1186/s13568-022-01395-w)
Supplement: Supplementary file 1 — Additional file 1: Figure S1. (+)-ESI-MS spectrum of compound 1, Figure S2. (+)-ESI-MS spectrum of compound 2, Figure S3. (+)-ESI-MS spectrum of compound 3, Figure S4. (+)-ESI-MS spectrum of compound 4 [file 13568_2022_1395_MOESM1_ESM.docx]

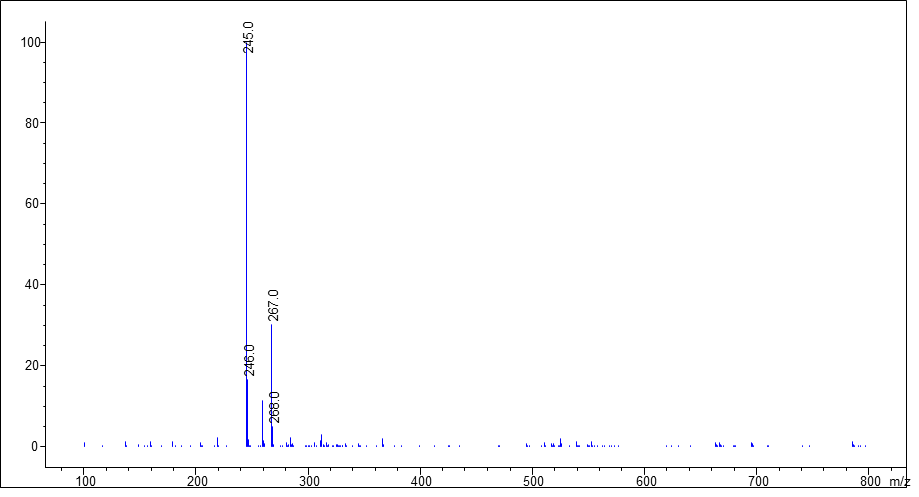


Figure S1. (+)-ESI-MS spectrum of compound **1**


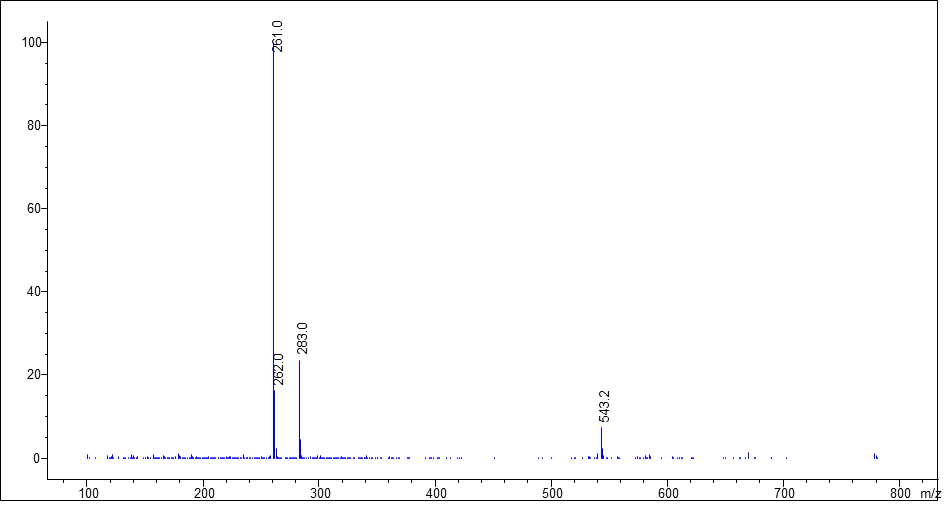


Figure S2. (+)-ESI-MS spectrum of compound **2**


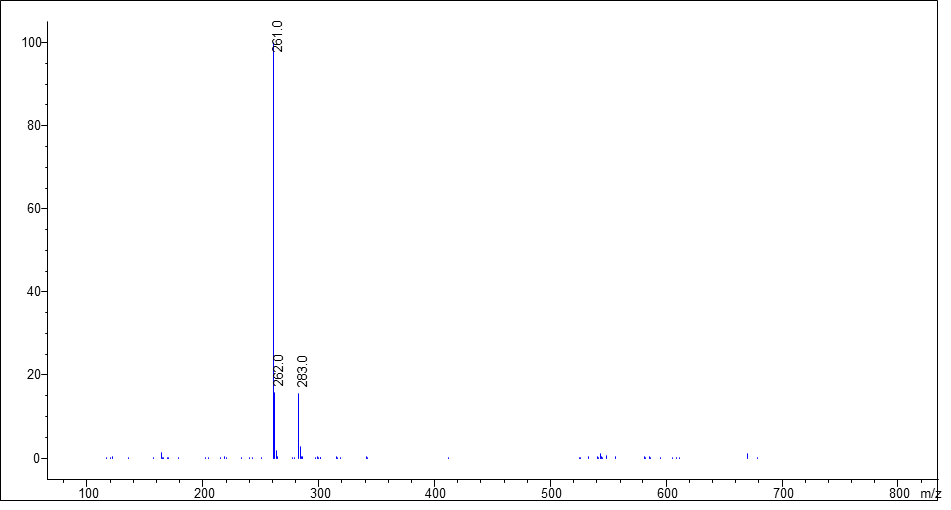


Figure S3. (+)-ESI-MS spectrum of compound **3**


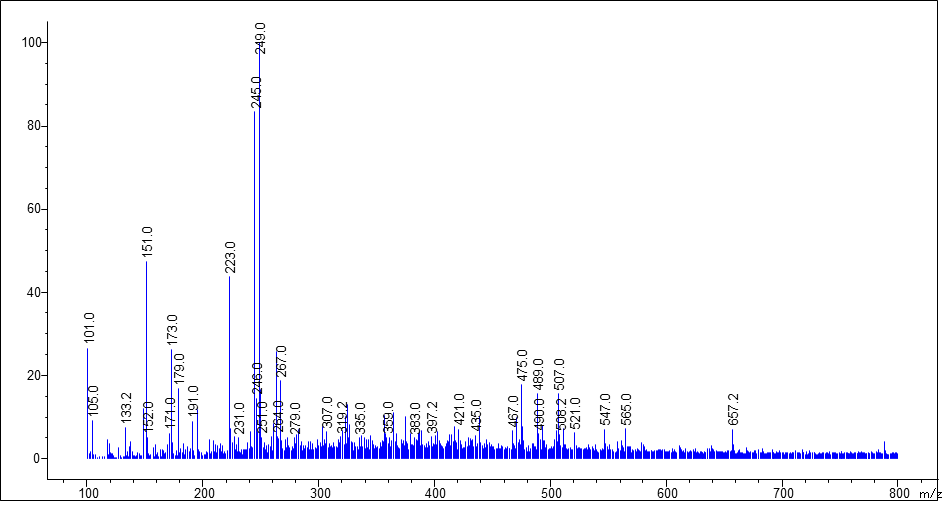


Figure S4. (+)-ESI-MS spectrum of compound **4**
